# In Silico Drug Design for Purinergic GPCRs: Overview on Molecular Dynamics Applied to Adenosine and P2Y Receptors

**DOI:** 10.3390/biom10060812

**Published:** 2020-05-26

**Authors:** Veronica Salmaso, Kenneth A. Jacobson

**Affiliations:** Molecular Recognition Section, Laboratory of Bioorganic Chemistry, National Institute of Diabetes and Digestive and Kidney Diseases, National Institutes of Health, Bethesda, MD 20892, USA; veronica.salmaso@nih.gov

**Keywords:** nucleoside, molecular dynamics, adenosine receptor, GPCR, extracellular loop, collective variable, membrane-bound proteins

## Abstract

Molecular modeling has contributed to drug discovery for purinergic GPCRs, including adenosine receptors (ARs) and P2Y receptors (P2YRs). Experimental structures and homology modeling have proven to be useful in understanding and predicting structure activity relationships (SAR) of agonists and antagonists. This review provides an excursus on molecular dynamics (MD) simulations applied to ARs and P2YRs. The binding modes of newly synthesized A_1_AR- and A_3_AR-selective nucleoside derivatives, potentially of use against depression and inflammation, respectively, have been predicted to recapitulate their SAR and the species dependence of A_3_AR affinity. P2Y_12_R and P2Y_1_R crystallographic structures, respectively, have provided a detailed understanding of the recognition of anti-inflammatory P2Y_14_R antagonists and a large group of allosteric and orthosteric antagonists of P2Y_1_R, an antithrombotic and neuroprotective target. MD of A_2A_AR (an anticancer and neuroprotective target), A_3_AR, and P2Y_1_R has identified microswitches that are putatively involved in receptor activation. The approach pathways of different ligands toward A_2A_AR and P2Y_1_R binding sites have also been explored. A_1_AR, A_2A_AR, and A_3_AR were utilizes to study allosteric phenomena, but locating the binding site of structurally diverse allosteric modulators, such as an A_3_AR enhancer LUF6000, is challenging. Ligand residence time, a predictor of in vivo efficacy, and the structural role of water were investigated through A_2A_AR MD simulations. Thus, new MD and other modeling algorithms have contributed to purinergic GPCR drug discovery.

## 1. Introduction

Molecular modeling has contributed to drug design and discovery for 40 years. Molecular docking and virtual screening techniques were initially employed to prefilter huge chemical libraries to reduce the burden of compounds and resources that are needed for in vitro testing. Structure-based drug design (SBDD) relies on the availability of the target macromolecule structure, as well as the commercial availability and informatic accessibility of now ultra-large libraries of small chemicals [1]. Moreover, the possibility of utilizing virtual libraries enables the investigation of chemical space in regions that have not yet been synthetically explored [2]. Over the last 20 years, hundreds of experimental structures have been solved for G protein-coupled receptors (GPCRs). These structures provide a snapshot of individual conformational states of the system, and they are useful for SBDD campaigns, with proven success in the identification of potential therapeutic compounds [3]. However, GPCRs in membranes are characterized by a high conformational plasticity, and molecular docking techniques starting from a static experimental structure neglect the target’s flexibility, as well as the membrane and solvent environment [4]. Recent software and hardware advances have extended the capabilities of another technique, molecular dynamics (MD), from an academic exercise to a useful drug discovery tool. The use of MD in the study of ligand-target complexes enables the inclusion of protein flexibility, with a better evaluation of the system stability. Furthermore, MD simulations can account for explicit solvent, ions, and membranes (in the case of membrane-bound proteins). Moreover, this might lead to the identification of protein’s druggable binding cavities, to the investigation of drug’s mechanistic pathways, and consequently to the computation of kinetic parameters [5]. In this review, we focus on recent MD studies of purinergic GPCRs, including adenosine receptors (ARs, also known as P1 receptors) and P2Y receptors (P2YRs) [6,7]. Both these receptor groups belong to the rhodopsin-like branch of Family A GPCRs. There are also purinergic ligand-gated P2X receptors (P2XRs) that are activated by ATP [8]. Structures have been determined for some of the seven P2XR subtypes, which act together as functional trimers, and modeling ligand interactions has been performed [9].

ARs are divided into four subtypes, all being activated by adenosine, but coupled to different G proteins. A_1_ and A_3_ ARs are coupled to G_i_ protein, and A_2A_ and A_2B_ ARs are coupled to G_s_ protein [10] (see Table 1). The four AR subtypes have been the subject of intense drug discovery efforts, leading to the identification of both agonists and antagonists [11,12]. Numerous A_2A_AR experimental structures have been released since 2008, as summarized in Table 2. In total, 49 structures for A_2A_AR have been solved and deposited in the Protein Data Bank (PDB) [13]: 40 antagonist-bound X-ray structures [14,15,16,17,18,19,20,21,22,23,24,25,26,27,28,29,30,31]; seven agonist-bound intermediate-state X-ray structures [32,33,34,35]; one agonist-bound active G protein-bound X-ray structure [36]; and one agonist-bound active G protein-bound cryogenic electron microscopy (cryo-EM) structure [37]. In addition, two X-ray structures of A_1_AR (antagonist-bound) have been reported [24,38], together with one agonist-bound active G protein-bound cryo-EM structure [39].

On the P2YR side, there are eight receptor subtypes, with P2Y_1_, P2Y_2_, P2Y_4_, P2Y_6_, and P2Y_11_Rs coupled to G_q_ (and G_i_ in the case of P2Y_2_ and P2Y_4_, and G_s_ in the case of P2Y_11_) and belonging to the P2Y_1_-like subfamily, and P2Y_12_, P2Y_13_ and P2Y_14_Rs coupled to G_i_ and belonging to the P2Y_12_-like subfamily. P2YRs are activated by diverse mono- and dinucleotides, as summarized in Table 1, with P2Y_1_, P2Y_12_, and P2Y_13_Rs activated by ADP, P2Y_2_ and P2Y_4_ by UTP and ATP (and GTP in the case of P2Y_4_), P2Y_6_ and P2Y_14_Rs by UDP (and UDP-glucose in the case of P2Y_14_), and P2Y_11_R by ATP [7]. P2Y_1_ and P2Y_12_Rs are the only P2YRs having an X-ray crystal structure deposited in the PDB. Two X-ray structures are available for P2Y_1_R [40], in complex with two different antagonists: MRS2500, a hydrophilic, nucleotide-like compound, bound to the orthosteric binding site within the transmembrane bundle, and BPTU, which is a hydrophobic diarylurea derivative, bound to an allosteric pocket outside the transmembrane bundle at the phospholipid interface. Two agonist-bound (2MeSADP and 2MeSATP) X-ray structures were solved for the P2Y_12_R [41], in addition to an antagonist-bound (AZD1283) structure where the conserved disulfide bond connecting TM3 and ECL2 was broken [42].

A_2A_AR is a cancer target, as antagonists block the local immunosuppressive effects of adenosine. P2Y_1_R antagonists are also potential antithrombotic and neuroprotective therapies. A_1_AR- and A_3_AR-selective agonists are potentially of use for treating depression and inflammation, respectively. A_2A_AR agonists and P2Y_14_R antagonists have anti-inflammatory properties.

The experimental structures of purinergic receptors have played a fundamental role in SBDD of agonists and antagonists for these receptors [43] and, lately, MD has gained an important role in this field. MD-based techniques have been applied to ARs and P2YRs to investigate association, dissociation, and activation processes, and, in some cases, with approximate free energy-profiles and binding kinetics (as association and dissociation rate constants). Other valuable applications include the simulation of phenomena such as allostery and exploration of the structural role of water. Before describing examples of MD applied to purinergic receptors, we provide an excursus on some of the main techniques that will be cited throughout this manuscript.

## 2. Excursus on MD Techniques

MD is a computational technique modeling the evolution of molecular systems in time at an atomistic level by approximating energy as a force field and solving Newton’s equations of motion. MD simulations can be used in combination with other structural techniques to assess the stability of complexes over time, to generate structural ensembles, and to identify putative cryptic pockets or allosteric sites. A timestep on the order of femtoseconds, corresponding to the fastest system motions (bond vibrations), is used; thus, MD is currently not suitable for the simulation of slow molecular processes [5]. The exploration of rare events, like receptor binding pathways and activation processes, requires a long timescale (hundreds of μs to ms), which are not easily achievable with classical MD simulations. Examples of long-timescale classical MD simulations are reported in literature, but they demand a huge computational cost and special resources [44]. To overcome this limitation, several techniques, called enhanced sampling techniques, have been developed in recent decades. These techniques are characterized by the addition of a bias potential to the system energy in order to enhance sampling and allow the system to jump with an increased frequency from one minimum to another within the energy landscape. Various enhanced sampling techniques exist, and they can be either based on collective variables (CVs), or not, where CVs are the reaction coordinates describing the process. Enhanced sampling techniques, after adding a bias potential to the original force-field energy of the system, require a subsequent phase of reweighting in order to recover the target system energy [45].

Metadynamics, Temperature-Accelerated MD (TAMD), Umbrella Sampling, Accelerated MD (aMD), and Gaussian Accelerated MD (GaMD) are some of the enhanced sampling MD techniques that will be considered in this review, as applied to ARs and P2YRs. Metadynamics consists of the decomposition of the system into one or more CVs, and of the application of a history-dependent bias potential that discourages the exploration of already visited states [46,47]. The system free-energy surface can then be reconstructed as a function of CVs while using reweighing approaches. TAMD was inspired by metadynamics, and it relies on dynamical collective variables to which an artificial high temperature is applied [48]. Umbrella sampling adds a harmonic compensating function to increase the sampling on high energy states [49]. aMD adds a boost potential to the true potential energy surface of the system to enable the overcoming of high energy barriers [50]. This method does not require a priori knowledge of the system to define CVs, but it suffers from poor energy reweighting due to the use of a high energetic bias. To overcome this limitation, GaMD was developed, where the bias potential is adaptively added to the potential energy surface following a Gaussian distribution, enabling better reweighting [51,52].

Classical MD simulations combined with adaptive sampling techniques can be employed to explore long time-scale events, by using multiple MD simulations sampling the search space on the basis of the collected data results [53]. Among these methods, Supervised MD (SuMD) was initially developed to simulate recognition of ligands by the A_2A_AR and consists of the supervision of classical MD simulations through a tabu-like algorithm, but without the introduction of any energetic bias [54,55]. A classical MD simulation is subdivided into different steps, during which the distance between the centers of mass of the ligand and of the receptor’s binding site is monitored. At the end of each step, the simulation cycles to a new step if the ligand has moved closer to the receptor; otherwise, the current step is restarted reassigning initial velocities. A SuMD algorithm modification recently enabled the simulation of ligand unbinding [56]. The algorithm was slightly modified in the criteria to define the success of a SuMD step (i.e., ligand moving away from the binding pocket’s center of mass) and by increasing the length of each SuMD step during the simulation.

## 3. MD Simulations Applied to Adenosine and P2Y Receptors

### 3.1. Postprocessing of Molecular Docking Poses

MD can be combined with molecular docking to predict a ligand binding mode at a target site, and to refine the consequent pose by taking into account the solvent contribution and the flexibility provided by all of the system’s degrees of freedom. For instance, associating MD simulations with molecular docking has recently been fruitful in rationalizing the AR-antagonist interactions for novel chemotypes identified through the virtual screening of a small library [57].

A methodological work illustrated the efficiency of a combination of molecular docking and MD simulations in reproducing the crystallographic pose of antagonists ZM241385 (ZMA), T4G, T4E, and caffeine, and of the agonist NECA at the A_2A_AR orthosteric binding site [58]. The computational pipeline consisted of molecular docking, pose clustering, MD simulations, and evaluation of the complex’s interaction energy fingerprints and interaction energy (electrostatic or hydrophobic) weighted by the root mean square deviation (RMSD).

**Figure 1:**
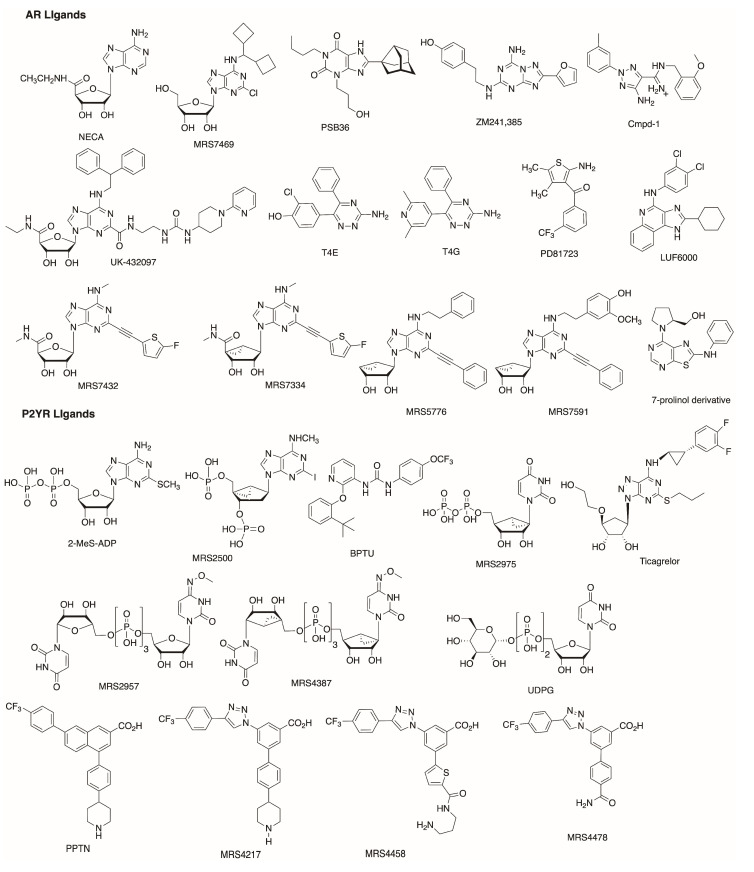
Structures of ARs and P2YRs (both orthosteric and allosteric) ligands discussed.

**Figure 2:**
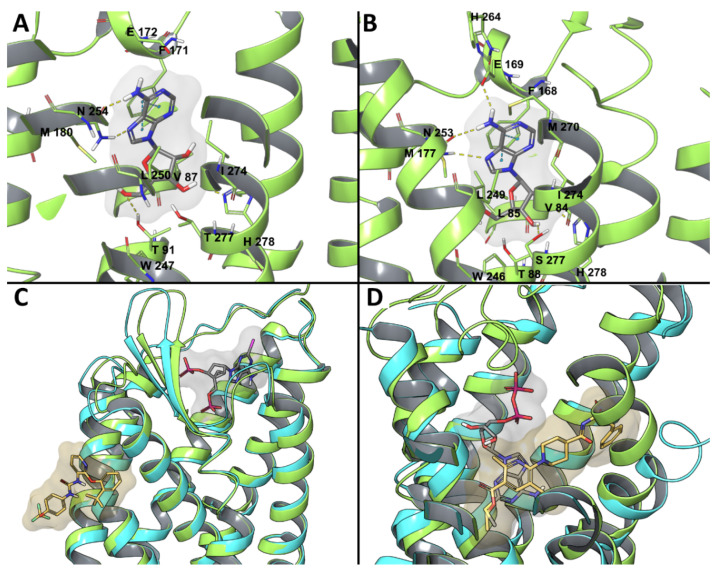
(**A**) cryogenic electron microscopy (cryo-EM) structure of the active-state A_1_AR (light green) bound to adenosine (gray) [39]. (**B**) X-ray structure of the intermediate-state A_2A_AR (light green) bound to adenosine (gray) [33]. (**C**) Superposition of the X-ray structures of MRS2500-bound and BPTU-bound P2Y_1_Rs [40]. MRS2500 is depicted by grays sticks and the corresponding receptor in light green, while BPTU is reported in orange sticks and the relative receptor in cyan. (**D**) Superposition of the X-ray structures of 2MeSADP-bound and AZD1283-bound P2Y_12_Rs [41,42]. 2MeSADP is depicted by gray sticks and the P2Y_12_R in light green, while AZD1283 is reported in orange sticks and the receptor in cyan.

Since 2008, when the A_2A_AR became the first AR experimental structure to be solved, it has been employed for numerous molecular modeling and computer aided drug design studies. More recently, two antagonist-bound X-ray structures [24,38] and a cryo-EM agonist-bound [39] structure of A_1_AR were released, increasing the reliability of computational works. A recent study was conducted to suggest a possible binding mode for the selective A_1_AR agonist *N*^6^-dicyclobutylmethyl-adenosine (MRS7469, Figure 1, hA_1_AR 2.14 nM, >2000-fold selectivity over the other AR subtypes) [59], while using the cryo-EM adenosine-bound A_1_AR structure (Figure 2A). A binding mode comparable to that of adenosine in the experimental complex was proposed for MRS7469, with the conserved N254^6.55^ being involved in a bidentate hydrogen bond with N7 and the exocyclic N^6^H, with a π-π stacking interaction between the adenine aromatic scaffold and F171^ECL2^, and with H-bonds between 2′-OH and H278^7.43^, 3′-OH and T277^7.42^, 5′-OH and H251^6.52^. All these interactions were maintained during 30 ns MD simulation, and the two cyclobutyl substituents occupied two hydrophobic clefts positioned among ECL2, TM5, and TM6 in one case and TM6 and TM7 in the other. Moreover, E172^ECL2^ and K265^ECL3^, which are separated by approximately 7 Å in the cryo-EM structure, narrowed their separation during the simulation, with a possible role in stabilizing the ligand bound state, as known for the E169^ECL2^-H264^ECL3^ salt bridge in the case of A_2A_AR, constituting a lid that caps the orthosteric binding site in several X-ray structures [19]. Moreover, an involvement of residues E172^ECL2^ and K265^ECL3^ in agonist binding was consistent with decreased binding affinity of NECA in the E172^ECL2^A and K265^ECL3^A mutant A_1_AR, with the mutation K265^ECL3^A being suggested by molecular modeling studies on a A_1_AR model [60].

A number of recent studies on the A_3_AR aided in the discovery of many nucleoside agonists and partial agonists, consistent with the observed structure activity relationship (SAR). Two prominent features of these A_3_AR ligands are the enhancement of affinity and selectivity provided by 2-arylethynyl substitution and ribose ring replacement with a chemically constrained (N)-methanocarba (bicyclo[3.1.0]hexane) ring system [61]. No experimental structure is available for A_3_AR, so molecular modeling studies are based on homology models built using other ARs structures as templates. In fact, homology modeling had previously proved to be an accurate approximation of the crystallographic structure of A_2A_AR (apart from ECLs and ICLs) [62], so it is a useful technique when the experimental receptor structure of interest is lacking. This is also valid for A_2B_AR, which also lacks an experimental structure, and whose molecular modeling studies have been recently summarized by Deb et al. [63]. Different A_3_AR models have been built in recent years [61], with early examples being based on rhodopsin structures [64], which were then replaced by A_3_AR based on A_2A_AR or, more recently, A_1_AR templates [65,66]. An antagonist-bound A_2A_AR-based A_3_AR model, for instance, was used to compare the behavior of C2-substituted adenosine and NECA derivatives though MD simulations. It was suggested that the C2 and 5′ substituents cooperate to maintain the interaction pattern of full or partial agonism that might be associated with an *anti* or *syn* glycosidic bond conformation [67].

A well established hypothesis, involving an outward movement of the upper portion of TM2 as compared to its position in A_2A_AR, was introduced in order to explain the binding mode of highly selective C2-arylethynyl substituted A_3_AR agonists [68]. The outward position of TM2 would enable an adenosine-like positioning of the agonists, avoiding clashes of the C2-substituent with TM2. The first models that were based on this hypothesis were built using an intermediate state agonist-bound structure of A_2A_AR, and opsin or β_2_-adrenergic receptor for TM2 [68]. Recently, a revised nucleoside-bound A_3_AR model was constructed, based on an intermediate state agonist-bound structure of A_2A_AR and an inactive antagonist-bound structure of A_1_AR for TM2, which is outwardly displaced from the TM bundle [69].

Human (h) and mouse (m) A_3_AR hybrid models that were built in this way were recently applied to rationalize the effective contribution of polar substituents on the *N*^6^-2-phenylethyl moiety of 4′-truncated (N)-methanocarba 2-phenylethynyl adenosine partial-agonists [69]. In particular, derivatization at the *N*^6^ position with a dopamine-like moiety increased both h and mA_3_AR binding affinity, with a particular increase in the case of mA_3_AR with respect to the unsubstituted *N*^6^-2-phenylethyl. A comparison between the binding modes of the *N*^6^-2-phenylethyl (MRS5776) and hydroxy/methoxylated *N*^6^-dopamine-like (MRS7591) derivatives was performed at hA_3_AR and mA_3_AR orthosteric sites through a combination of molecular docking and classic MD simulations. The compounds’ adenosine-like scaffold similarly bound the two receptors to the binding mode adenosine or other agonists assumed in the experimental structure of A_1_AR or A_2A_AR (Figure 2A,B) [32,33,34,35,36,37,39]: a bidentate H-bond between N250^6.55^ (N251^6.55^ at mA_3_AR) and the ligands’ N7 and exocyclic N^6^H, a π- π stacking between the adenine aromatic scaffold and F168^ECL2^ (F169^ECL2^ (m)), and the H-bonds of hydroxyls 2′ and 3′ with H272^7.43^ and S271^7.42^ (respectively H273^7.43^ (m) and S272^7.42^ (m)), which were less persistent during MD. Contacts with residues that are involved in agonist binding and/or receptor activation according to site-directed mutagenesis (SDM) studies [70,71,72] were observed during the simulations: L90^3.32^ (L91^3.32^ (m)), T94^3.36^ (T95^3.36^ (m)), M177^5.38^ (M178^5.38^ (m)), W243^6.48^ (W244^6.48^ (m)), L246^6.51^ (L247^6.51^ (m)), I268^7.39^ (I269^7.39^ (m)), in addition to the aforementioned residues. Together with this, MRS7591 transiently interacted with residues of ECL2 and ECL3 in both hA_3_AR and mA_3_AR simulations, possibly explaining the higher affinity of this compound for the receptor in comparison to MRS5776. The enhanced effect in the case of mA_3_AR could be rationalized by the generally higher polar character of the extracellular regions of this receptor in comparison to the human homologue.

In the aforementioned study, the 2-arylethynyl moiety pointed toward the outwardly displaced TM2, as expected for 2-substituted nucleosides. However, an alternative binding mode was recently proposed for a series of 2-arylalkynyl-adenine hA_3_AR antagonists, where the lack of the ribose moiety enabled the compounds to occupy the orthosteric site upside down, with a bidentate H-bond between the key N250^6.55^ and the adenine N3 and N9 positions, and the 2-arylalkynyl group hosted by a hydrophobic pocket between TM5 and TM6 [73]. Thus, the adenine moiety does not need to persist in the same orientation as in the nucleoside when the ribose or pseudoribose is absent. 

**Figure 3:**
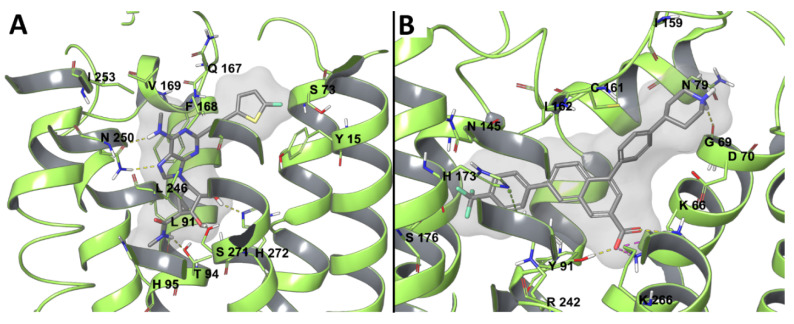
(**A**) Docking pose of MRS7334 (gray) [74] at an A_3_AR model (light green) [69]. (**B**) Docking pose of PPTN (gray) at a P2Y_14_R model (light green) [78].

Another study was recently conducted using the aforementioned hybrid hA_3_AR model [69] to compare the bound state of a ribose and a (N)-methanocarba agonist analogue, namely MRS7432 and MRS7334 (Figure 3A) [74]. The (N)-methanocarba ligand had higher affinity for the receptor as compared to the ribose analogue, following the typical SAR for A_3_AR. During MD simulations, the two ligands behaved similarly in maintaining the typical pattern of interactions described above. The puckering parameters were computed for the two ligands in the bound state during time and they were compared to the behavior of the compounds in a free state. The ribose pucker of MRS7432 experienced a wide range of conformations ranging from (N) to (S) and passing through (E) in the free state, but the conformational space it explored in the bound state appeared to be reduced. The lack of this phenomenon in the case of the constrained (N)-methanocarba MRS7334 suggested that an entropy loss upon binding might explain the lower affinity of ribose-containing agonists as compared to conformationally constrained (N)-methanocarba.

The conformational constraint induced by the ribose ring substitution with a methanocarba was also applied to P2YR agonists. Dramatically, the (N)-methanocarba substitution prevented binding, while the isomeric (S)-methanocarba modification of UDP (MRS2975) increased its P2Y_6_R affinity. Dinucleotide analogues, particularly dinucleoside triphosphates, are also known to activate the P2Y_6_R. A series of alkyloxyimino-pyrimidine dinucleotides presenting a (S)-methanocarba or a (N)-methanocarba at either one or both nucleoside moieties showed that the highest potency is reached when one nucleotide (MRS4387) was constrained in the (N) and the other in the (S) conformation [75]. The reference compound, MRS2957, bearing two unconstrained ribose rings, was docked to a P2Y_6_R homology model that was built using the chemokine receptor 4 (CXCR4) as a template, and the complex was subjected to MD simulation. One uridine (called proximal) was hosted by a pocket between the conserved TM3-ECL2 disulfide bond and TM7 and the other (the distal one) lay above the orthosteric binding site. The proximal uridine showed an (S) pucker for the major part of a 100 ns MD simulation, while the distal uridine spent most simulation time in a (N) pucker, consistently with the experimental data.

Among P2YRs, the availability of crystallographic structures for P2Y_1_R and P2Y_12_R (Figure 2C,D) in recent years increased the reliability of molecular modeling studies on this subfamily of receptors. A molecular modeling study on P2Y_1_R combining docking and MD simulations recapitulated the SAR of a series of 100 nucleotide-like biphosphates that were structurally related to MRS2500 [76]. This study highlighted a steric limit for C2-alkyl substituents that cannot fit the hydrophobic pocket defined by ECL3 and N-term and, thus, are solvent exposed, with a balance provided by H-bond capable moieties. The intolerance of C8 substitutions was justified by the incompatibility with the *anti*-glycosidic bond present in the experimental MRS2500 conformation (Figure 2C) and maintained during the MD simulation. The agonist 2MeSADP showed a preferred (N) conformation during the MD simulation, which gave rationale to the efficient constraint that was provided by the (N)-methanocarba in MRS2500.

The therapeutic relevance of P2Y_14_R for inflammatory diseases has raised the interest in antagonist development for this receptor subtype, which lacks an experimental structure. A P2Y_14_R homology model was built on the basis of an agonist-bound P2Y_12_R structure, belonging to the same P2Y_12_-like subfamily [77]. The docking of UDP to the P2Y_14_R model provided a possible *syn*- or *anti*-glycosidic bond conformation, and MD simulation was used to discriminate the two, selecting the *anti*- as being more likely, in agreement with the pose of 2MeSADP bound to the P2Y_12_R X-ray structure. The substitution of C194^5.43^ of P2Y_12_R with F191^5.43^ in P2Y_14_R was hypothesized as a selectivity filter allowing pyrimidine and not purine binding. UDP occupied a pocket that was delimited by TMs 3, 4, 5, 6, and 7, with the pyrimidine base making a π-π interaction with Y102^3.33^, the ribose ring involved in H-bonds with the side chains of H184^5.35^ and K176^ECL2^ and the backbone carbonyl of C94^3.25^. The same model was then refined to be employed for SBDD of P2Y_14_R antagonists [78,79]. The prototype antagonist used for the refinement was the highly active and selective antagonist PPTN, which was docked with an induced fit procedure and subjected to 10 ns MD simulation, showing modification in the model geometry (Figure 3B). In particular, TM7, as well as ECL1 and ECL2, moved outward from the TM bundle, and the axis of TM2 slightly bent toward TM3. PPTN was then re-docked and the complex stability was assessed during MD simulation; the compound interacted through its carboxylate group with K77^2.60^, Y102^3.33^, and K277^7.35^ by means of H-bonds (and ionic interactions in the case of charged residues), and through the piperidine nitrogen with the backbone carbonyl of I170^ECL2^ (replacing the interaction with G80^2.63^ observed in the docking pose) [78]. The poor solubility and low oral bioavailability of PPTN has necessitated the development of new P2Y_14_R antagonists, and the same protocol made for docking and MD refinement was used for investigating a bioisosteric substitution of the scaffold’s naphthalene portion. Two alternatives were considered, an alkyne and a triazole substitution, with the latter maximizing the pattern of interactions during MD simulations, in agreement with its higher in vitro affinity for the receptor when compared to the alkyne. While the alkyne derivative lost the H-bond with Y102^3.33^ and had a higher root mean square fluctuation (RMSF) as compared to the precursor, the triazole derivative had a lower RMSF and it was additionally stabilized by a π-π interaction between the triazole and Y102^3.33^ and between the 4-(4-trifluoromethyl)-phenyl group and His184^5.36^, and by a π-cation interaction between the triazole and R253^6.55^ [78]. In agreement with this, MRS4217, a triazole analogue of PPTN, was considered to be the lead compound for a series of analogues, whose SAR, in agreement with molecular modeling results, highlighted the requirement of the carboxylate and of a hydrophobic substituent preferentially in *p*- position on the 4-aryl group attached to the triazole [78,79]. Different substituents of the 5-(4-piperidin)-phenyl moiety were analyzed trying to maximize interactions with ECLs: the replacement of the phenyl ring with a thiophene and of piperidine with a propylamine were suggested by the docking results. A comparison of the two analogues with an amide or sulfonamide linker showed a similar behavior in MD simulations, with an H-bond between the carboxylate and Y102^3.33^, and between the amine and G80^2.63^, a π−π stacking between the 4-(4-trifluoromethyl)-phenyl moiety and H184^5.36^ and between the triazole and Y102^3.33^. Two salt bridges between the carboxylate and K77^2.60^ and K277^7.35^ were present with the sulfonamide compound, but they occurred between the carboxylate and K77^2.60^ and R253^6.55^ with the amide ligand. The amide compound (MRS4458, the most potent of the series) was more rigid and fitted in a slightly deeper position, granting an additional ionic interaction between the carboxylate and R253^6.55^. Another compound (MRS4478), with a 5-(4-carboxamide)-phenyl substituent, showed a promising binding affinity to P2Y_14_R and it behaved similarly to the other two ligands during MD simulations, with, as only the difference, an interaction between the amide and N90^3.21^ [79].

An advanced MD technique can be applied in order to estimate ligand-target binding affinity, starting from ligand-target complexes that originate from either molecular docking or experimental structures. This technique is called Free Energy Perturbation (FEP) and it consists of a statistical mechanics method computing the free energy change related to an alchemical process, like the transformation of a ligand into another, giving the relative binding free energy of the two compounds to the same target [80]. In the field of purinergic receptors, FEP was used to compare a thiazolo [5,4-d]pyrimidine partial agonist having a 7-prolinol substitution to its antagonist analogue lacking the prolinol’s 2-hydroxymethylene moiety. The simulations of the compounds docked to the binding site of an inactive and an active-like A_2A_AR structures showed that the loss of the 2-hydroxymethylene group was more unfavorable in the active-like state, in agreement with the potency reduction of the antagonist [81]. Prospective FEP+ (a FEP modification [82]) studies predicted a highly potent A_2A_AR antagonist in the nanomolar range, with a ten-fold increase in binding affinity as compared to the parent compound [83]. A different application of FEP enabled the exploration of the influence of alanine-scanning in agonist (NECA) and antagonist (ZMA) binding to A_2A_AR, with a good correlation with the experimental data [84]. In a study on *N*-(4,6-diarylpyridin-2-yl)acetamides as A_3_AR antagonists, FEP was used to rationalize the reduction in binding affinity, due to a bioisosteric substitution of N1 with CH and to suggest a likely binding mode [85]. Moreover, examples of using FEP in combination with Fragment-Based Lead Discovery (FBLD) are reported, where MD and FEP played an important role in fragments optimization as A_2A_AR binders [86,87]. 

### 3.2. Activation and Deactivation

Many MD studies have been conducted in order to investigate one of the most intriguing mechanistic processes related to GPCRs, activation, and deactivation. A number of common structural motives, called “microswitches”, are known to play a key role in GPCR activation and, among them: the ionic lock between R^3.50^ of the conserved E/DR^3.50^Y motif on TM3 and E^6.30^ on TM6 typical of the inactive state, the “rotamer toggle switch” or “transmission switch” characterized by a different conformation of W^6.48^ according to the receptor activation state, and the “tyrosine toggle switch” characterized by a conformational rearrangement of Y^7.53^ of the conserved NPxxY^7.53^ motif [88].

The formation and maintenance of a salt bridge between R102^3.50^ and E228^6.30^, the so-called ionic lock, typical of GPCR inactive states, was observed in different works simulating pseudo-apo states coming from antagonist-bound structures [89,90]. An alternative salt bridge involving E228^6.30^ and R105^3.55^, together with an outward movement of the TM5 intracellular portion, was observed in the X-ray structure of A_2A_AR bound to a triazole-carboximidamide antagonist (Cmpd-1) [21], which was recently verified through adaptive sampling MD simulations and ^19^F-NMR spectroscopy [91]. 

Together with the R102^3.50^-E228^6.30^ “ionic lock”, the rotameric transition of W^6.48^ (“toggle switch”), of the conserved CW^6.48^xP motif, is considered to play a key role in the GPCR activation process, and a conformational rearrangement was observed for W246^6.48^ in a pseudo-apo A_2A_AR MD simulations coming from inactive antagonist (ZMA)-bound structures [89]. Moreover, simulations in the order of μs showed that agonist (NECA) binding to A_2A_AR induced a conformational rearrangement of W246^6.48^. This movement allowed for water flow through the transmembrane helical (TM) bundle [92], which provoked a rotational switch of residue Y288^7.53^ (of the conserved NPxxY^7.53^ motif), an outward movement of TM6 and inward movement of TM7, causing an opening of the receptor’s internal portion, which could enable G protein binding [93]. The concerted movement starting from the toggle switch to the TM6 outward movement upon agonist binding is in agreement with a previous study, where TM6 appeared to assume an active-like, inactive-like, and intermediate conformation in agonist-bound, antagonist-bound, and pseudo-apo simulations, respectively [94]. An adaptive sampling study combined with Markov State Models computation lead to the simulation of the activation energy landscape of an apo A_2A_AR, which could be approximated by four predominant states. These four states are: (1) inactive antagonist-bound like state with the ionic lock between R102^3.50^ and E228^6.30^ and TM6 in proximity to TM3; (2) the apo intermediate-inactive state with disruption of the ionic lock and its own peculiar TM6 conformation; (3) the agonist-competent state, with TM6 moved outward and Y288^7.53^ pointing toward TM5 preventing G protein binding; and, (4) the active state, with TM6 pointing outward and an overall more open intracellular portion of the receptor. The active state was minimally populated in the explored activation energy landscape, in agreement with the concept of the basal activity of GPCRs [95]. A common character of all simulations of A_2A_AR activation/inactivation processes is an outward TM6 movement during receptor activation upon agonist binding. This phenomenon was experimentally confirmed by the experimental structures of a fully active A_2A_AR bound to agonist NECA and an engineered G protein, where the TM6 intracellular portion is 14 Å away from the central axis of the TM bundle, and TM5 and TM7 slightly deviate [36,37]. In another study, the MD simulations showed decreased entropy of the extracellular portion when agonists (adenosine or NECA) bound an intermediate-active or active (G protein-bound or free) receptor rather than the inactive one. In parallel, a flexibility increase in the intracellular portion was observed when agonists bound the intermediate-active or G protein-free active state as compared to the inactive one, but stabilization was observed in the G protein-bound active state. The agonist’s geometric stability was higher, the orthosteric pocket volume was reduced, and the pattern of ligand-receptor interactions stabilized in the intermediate-active and G-protein bound active receptor. All these observations well supported the gradually increased computed agonist binding affinity going from the inactive, the intermediate-active and G-protein bound active receptor [96].

A_2A_AR has been, for a long time, the test case for many molecular modeling studies as an AR prototype, thanks to the early availability of many X-ray crystal structures. More recently, for the A_1_AR two inactive antagonist-bound structures [24,38] and a cryo-EM active agonist-bound structure [39] were released, so more reliable molecular modeling studies are now possible. Recently, GaMD investigated the flexibility profile of the active adenosine-bound and inactive PSB36-bound A_1_AR [97]. In both simulations, conformations of the ECL2 region and the intracellular end of TM6 fluctuated, with higher flexibility of these receptor portions and of the intracellular end of TM5 in the active state.

The A_3_AR activation mechanism has been recently investigated through the simulation of three constitutively active mutants (CAMs), R108K^3.50^, R108A^3.50^, and A229E^6.34^, and a wild-type structures, modelled on the inactive and active (G protein bound and unbound) X-ray structures of A_2A_AR. The salt-bridges D107^3.49^-R108^3.50^ (with the two residues belonging to the conserved E/DR^3.50^Y motif on TM3) and E255^6.30^-R111^3.53^ characterized the inactive state, while salt-bridges D107^3.49^-R111^3.53^ and E255^6.30^-R205^5.66^ characterized the active one. Together with this, different conformations of W243^6.48^ (microswitch) were observed in the active and inactive states, an interaction between a Na^+^ ion and D58^2.50^ characterized the inactive state, and an increased flow of water molecules characterized the active one [98].

A study on agonist- and antagonist-bound structures of P2Y_1_R was reported, simulating the receptor bound to BPTU (in its extrahelical binding site), MR2500, and ADP through long classical MD simulations [99]. An ionic-lock between D204^ECL2^ and R310^7.39^ was present in both antagonist-bound P2Y_1_R simulations, while it was broken in the agonist-bound simulation; these residues were confirmed to be essential for activation by SDM studies. ADP was predicted to bind the receptor extracellular portion, similarly to MRS2500, but the binding pocket in the agonist-bound state appeared to be larger than in the antagonist-bound one. The increased pocket volume was due to a continuum of water flux that spanned the TM bundle with a consequent rotameric switch of Y324^7.53^ (of the conserved NPxxY^7.53^ motif), and a shift of the cytoplasmic region of TM3, TM6, and TM7 that enlarged the intracellular cavity for G protein binding. The presence of the ionic lock between D204^ECL2^ and R310^7.39^ was also confirmed for the BPTU-bound P2Y_1_R in another study, but interaction between D204^ECL2^ and K280^6.55^ was instead observed as a characteristic of 3′,5′-biphosphate ligands [76]. Differently, in the same study, simulations of the 2MeSADP-bound receptor (built in place on the basis of MRS2500) showed a deviation of K280^6.55^ from the binding site and an interaction with Y214^5.35^, H132^3.33^, Y136^3.37^, in agreement with the reduced ligand affinity or potency in the case of K280^6.55^A, H132^3.33^A, and Y136^3.37^A mutations [100]. The same study confirmed also that the water molecule diffusion within the TM bundle was enhanced in the case of the pseudo-apo or nucleotide agonist-bound form of P2Y_1_R, while it was reduced in the MRS2500-bound receptor simulation.

A D204^ECL2^-R310^7.39^ homologue ionic lock was also observed in the models of P2Y_2_R and P2Y_4_R built on the inactive P2Y_1_R template, with D185^ECL2^-R292^7.39^ and D187 ^ECL2^-R292^7.39^ being present in P2Y_2_R and P2Y_4_R, respectively. Agonists’ phosphate groups are predicted to interact with these residues, suggesting an activation mechanism that involves the disruption of the ionic lock, similarly to P2Y_1_ [101,102,103,104].

Another study on the agonist 2MeSADP-P2Y_1_R complex suggested a different binding site for the ligand, different from MRS2500, but comparable to the conformation of the same ligand in the X-ray structure of 2MeSADP-P2Y_12_R and compatible with SDM data [105]. This binding mode was obtained by docking the ligand in an ECL2 deprived inactive P2Y_1_R and subjecting the complex (after restoring ECL2) to aMD. The ligand was found to participate in π-π stacking and a cation-π interaction with H132^3.33^ and K280^6.55^ through the aromatic purine ring, a H-bond with Y222^5.43^ through the exocyclic amino group, a H-bond with Y136^3.37^ and T221^5.42^ through N1, and ionic and polar interactions with R128^3.29^, R287^6.62^, R310^7.39^, K280^6.55^, and Y306^7.35^ through the pyrophosphates. Moreover, the agonist-bound receptor underwent an outward movement of TM6 in the intracellular extremity, a S146^3.47^-Y237^5.58^ H-bond rupture, with the formation of a Y237^5.58^-V262^6.37^ (backbone O) H-bond and rotamer modification of F269^6.44^, which were not observed in the simulation of the MRS2500-bound inactive receptor in the same work.

### 3.3. Association Process

In the last years, a paradigm shift has been accompanying drug discovery, with increased attention to kinetic parameters in addition to binding affinities. For this reason, several methods have been developed to compute binding rate constants and study binding pathways [106].

The binding pathway of high affinity antagonist ZMA to the A_2A_AR was explored using well-tempered metadynamics [107]. The bound state showing lowest energy presented a salt bridge between E169^ECL2^ and H264^ECL3^, and its calculated binding free energy compared well to the experimental one. A metastable binding site was found in a vestibule that formed by ECL1 and ECL2 and the tips of TM1, TM2, and TM7, with two of the engaged residues, S67^2.65^ and L267^7.32^, known to increase the residence time of ZMA without influencing binding affinity if mutated to Ala. A transient low populated state showed the interaction with K153^ECL2^, which is reported to decrease the dissociation rate of ZMA if mutated into Ala. 

SuMD simulations investigated the involvement of the A_2A_AR ECLs in the initial stages of the recognition of various ligands, where the binding event of ZMA, T4G, and T4E (reaching a bound state comparable to the crystallographic one in approximately 60 ns, 65 ns, and 110 ns of SuMD simulation time, respectively) was anticipated by contacts with residues of ECL2 and ECL3 [54]. In the same way, adenosine (as well as its metabolite inosine) approached A_2A_AR breaking the ionic interaction between E169^ECL2^ and H264^ECL3^ (in agreement with the rupture of this interaction in previously reported pseudo-apo simulations of A_2A_AR [89]) [108,109]. This binding occurred through two main pathways, one involving in the first stages of recognition ECL2, ECL3 and the tips of TM5 and TM6 (similarly to what observed also for the agonist NECA [55]), and the other just ECL2. In the orthosteric binding pocket adenosine was observed to explore different bound states with the ribose pointing toward either the intracellular or the extracellular region. A bound state that was similar to the crystallographic one was only reached in the G protein-bound receptor simulation, supporting the hypothesis that adenosine binds and stabilizes through conformational selection for the receptor active state. A speculative 2:1 binding stoichiometry of adenosine to A_2A_AR was also suggested using SuMD simulations, supposing a kinetic rather than a thermodynamic driven event [110].

A recent application of SuMD to a hybrid A_2B_AR-A_2A_AR model contributed to the investigation of the role of ECL2 in defining the different binding affinity of A_2A_AR and A_2B_AR for the endogenous agonist adenosine [111]. The two AR subtypes have a high sequence identity (59%), with the bigger differences relative to ECL2 (34%, with A_2B_AR ECL2 longer than that of A_2A_AR), but A_2A_AR has high affinity for adenosine (nanomolar), while A_2B_AR has low affinity (micromolar). The introduction of A_2B_AR ECL2 in A_2A_AR reduced the affinity for adenosine by ~20-fold. A hypothetical explanation was provided through a SuMD trajectory showing a metastable binding site at the ECL2, which seemed to be energetically favored in comparison to the orthosteric site.

The recognition of adenosine by A_3_AR was also simulated with SuMD, observing also in this case a first contact with ECL2 and ECL3, and a final stabilization in the orthosteric binding site in 20 ns, with contacts with the conserved N250^6.55^, F168^ECL2^, W243^6.48^, and I269^7.39^ [112].

An application of SuMD to the P2Y subfamily was performed while simulating the antagonist ticagrelor’s P2Y_12_R binding pathway: the ligand was not able to reach the putative orthosteric binding site (supposedly comparable to the crystallographic 2MeSADP one), but it was stuck in metastable binding sites engaged in numerous polar and ionic interactions with residues belonging to ECL2 and the tips of TM5, TM6, and TM7 [113]. Differently from the ARs scenario, this could support the idea of a possible plasticity of the extracellular region of P2Y_12_R, which should be able to recognize different ligands.

The simulation of the approach of ADP, placed initially about 15 Å from the orthosteric binding site, to P2Y_1_R was studied with long classical MD simulations [99]. ADP was predicted to bind similarly to the antagonist MRS2500, in the extracellular vestibule, with the purine base undertaking a π-π interaction with Y303^7.32^, the ribose interacting with the same residue through a H-bond network that is mediated by water, and the pyrophosphate interacting through charge interactions with K41^1.41^, K46^1.46^, R195^EL2^, and R287^6.38^, consistent with SDM data [40,114].

The antagonist BPTU’s binding process from the bulk solvent to its extrahelical P2Y_1_R binding site was simulated through well-tempered metadynamics, and three stages were identified from the Free Energy Surface: the ligand entered the phospholipid bilayer, diffused in the bilayer, and interacted with I118^ECL1^ and F119^ECL1^ making contacts with ECL2, and finally reached the final position in the lipid-receptor binding site, with a RMSD < 2 Å from the crystallographic pose, with two single ligand H-bonds with L102^2.55^ [115].

### 3.4. Allostery

GPCRs inherently function as allosteric engines, because a conformational change in one location (extracellular) can affect the conformation at a remote location, enabling coupling to intracellular messengers. Different partners induce a population shift in the conformational equilibrium of GPCRs [116]. Unfortunately, structural information on GPCRs bound to allosteric modulators is limited, so the description of allostery from a structural perspective relies especially on MD simulations, which helps to fill a gap in this field.

GaMD recently investigated the binding of positive allosteric modulators (PAMs) to A_1_AR. The spontaneous binding of 2-amino-3-benzoylthiophenes, PD81723 and VCP171, was simulated in the presence of NECA that was bound to the orthosteric binding site [117]. Both PAMs bound a site in ECL2, consistent with mutagenesis data, and they were observed to stabilize the binding of NECA, especially through the formation of a salt bridge between E172^ECL2^ and K265^ECL3^. In fact, NECA explored a larger conformational space and it could also dissociate from the receptor in the absence of the PAM in the ECL2 cleft. The involvement of ECL2 in the binding of allosteric ligands seems promising for addressing the selectivity issue in AR drug discovery, with this receptor region showing low sequence identity in comparison to the TM bundle among the receptors and structural diversity in the experimental A_1_AR and A_2A_AR structures. Moreover, E172^ECL2^ appeared to be relevant in A_1_AR binding of both PD81723 and VCP171 in a previous study employing a receptor homology model, and that showed a pharmacological reduction of the allosteric ligand affinity for the E172^ECL2^A mutant receptor [118].

Locating the binding site of structurally diverse allosteric modulators, such as PAM LUF6000, has been challenging in most cases, even when SDM data are available. SuMD was employed to investigate the possible allosteric pathways of LUF6000 to A_3_AR in presence of adenosine bound to the orthosteric pocket. Two allosteric mechanisms were observed: binding to ECL2 with the induction of conformational changes able to stabilize adenosine deeper in the orthosteric pocket or capping the orthosteric pocket to form a ternary complex with adenosine and the receptor [112].

Among the well-known allosteric modulators of GPCRs, sodium is widely reported with its crystallographic conserved allosteric binding site among GPCRs [119]. An MD study was performed to compare the influence of the ion on an inactive and intermediate-active conformation of A_2A_AR in the presence of antagonist ZMA or the agonists UK432097 and NECA [120]. In the crystallographic ZMA-bound inactive structure, the ion was coordinated by D52^2.50^, S91^3.39^, and water molecules, mediating interactions with W246^6.48^, N280^7.45^, T88^3.36^, and S281^7.46^. The ionic interaction with D52^2.50^ was monitored and remained stable in all the ZMA-bound simulations, but two alternate states were observed for sodium interacting either with S91^3.39^ or N280^7.45^. In the presence of sodium and/or the antagonist ZMA, the conformation of W246^6.48^ remained unaltered, while in the pseudo-apo receptor without sodium, a rotameric transition was observed for that residue (the already mentioned “toggle switch”), which seemed connected with a rotation of N280^7.45^. From the simulations of the intermediate-active state of A_2A_AR, bound to an agonist (UK432097 or NECA) or in the pseudo-apo form, binding of sodium and agonists were observed to be mutually exclusive: the ion was unstable, since it escaped from its binding site in some simulations or, in others, stayed in place, but a conformational change of TM7 moving apart from TM3, resembling the inactive state, occurred. Thus, the MD simulations were in agreement with experimental data highlighting the reduction of NECA and enhancement of ZMA binding in the presence of sodium, with a stabilization of the receptor inactive state. A study analyzed the effect of mutations of residues of the first and second sodium coordination shells, in particular, of D52A^2.50^, S91A^3.39^, W246A^6.48^, N280A^7.45^, and N284A^7.49^, by means of in vitro radioligand displacement studies and MD simulations [121]. In the case of the D52A^2.50^ mutant, sodium moved from its site to a pocket that formed by E13^1.39^ and H278^7.43^, giving a possible explanation to the enhancement of NECA binding at high concentration of NaCl for this mutant receptor. Since sodium can bind to a different pocket, the binding of the ion and the agonist are not exclusive. S91A^3.39^, W246A^6.48^, N280A^7.45^, and N284A^7.49^ did not affect the ion mobility, but the interactions with the three coordinating residues were lowered during the simulations, and the mutations S91A^3.39^, W246A^6.48^, and N280A^7.45^ had a modest (or none in the case of N284A^7.49^) in vitro effect on NECA binding. SuMD simulations were performed in order to investigate the binding pathway of sodium from bulk solvent toward its allosteric pocket [122]. The ion reached the binding site of the pseudo-apo A_2A_AR coming either from an antagonist (ZMA)-bound structure or from an agonist (adenosine)-bound one, but, in the second case, a TM domain rearrangement was required, with TM2 and TM3 increasing their distance and TM7 moving outward. A contribution of W246^6.48^ was observed in mediating the final transition of the ion to the allosteric site during the binding pathway. Furthermore, SuMD enabled the investigation of adenosine recognition from the sodium-free intermediate-active A_2A_AR, but the simulation was unfruitful in most replicates of the sodium-bound inactive receptor.

In addition to the well characterized allosteric effect of sodium, a combination of ^19^F-NMR experiments and MD simulations showed that a high concentration of calcium and magnesium ions acts as A_2A_AR positive allosteric modulators. MD simulations in large excess of the ions highlighted the formation of stable cation-bridged interactions (E151^ECL2^-E161^ECL2^, E151^ECL2^-E169^ECL2^-D170^ECL2^, and D261^ECL3^-D170^ECL2^) with a consequent compaction of the receptor’s extracellular portion, which was allosterically propagated to an opening of the intracellular G protein-binding cavity. This would facilitate a receptor allosteric activation, further enhanced by the cations’ interaction with E228^6.30^ disfavoring the ionic lock [123].

Lipid allostery on A_2A_AR contributes to an active-like conformation, as shown using unbiased, long (µsec) MD simulations [124,125]. In addition, receptor-cholesterol interactions have been explored with MD simulations [126,127,128], including the observation of cholesterol entering the receptor, going beyond its solely allosteric effect [129].

### 3.5. Dissociation Process

In recent years, the paradigm of evaluating pharmacological activity in terms of thermodynamic binding parameters has been accompanied by a perspective on ligand residence time [130]. A longer residence time, i.e., slower dissociation rate, is thought to be predictive of in vivo efficacy. Thus, MD simulations recently investigated this phenomenon.

A method, called Smoothened Potential MD, has been proposed to estimate the relative k_off_ ranking within a series of congeneric compounds with respect to a reference. Scaled potentials are applied in order to enhance the transition probability between different energy minima in MD simulations, without the need of CVs. This method was applied to a series of triazine-based antagonists in complex with the A_2A_AR, which were correctly ranked with a correlation coefficient of 0.95 [131].

In the same year, the aMetaD method was developed combining adiabatic-bias MD and well-tempered metadynamics to rapidly simulate dissociation events [132]. aMetaD evaluates the maximum energy (TS energy) bias that is needed to move a ligand from the starting energy basin to the other, giving a residence time score (RTscore). RTscore was able to discriminate the residence time difference between A_2A_AR-bound compounds 4e and 4a.

A recent dissociation study focusing on A_2A_AR developed a method employing ensemble-based steered MD (SMD) to determine the residence time on the basis of a different water-ligand interaction energy (ΔE_LW_) between unbound and bound ligand states [133]. The calculated change in water-ligand interaction energy correlated well (R^2^ = 0.79) with the experimental residence time for 17 A_2A_AR ligands. While antagonist ZMA was extracted from the receptor, thirteen residues made intermediate contacts with the ligand during the dissociation pathway. Eight of these residues had been mutated into Ala in a previous study, and seven of them had kinetic and/or thermodynamic effects: I66^2.64^A, S67^2.65^A, K153^ECL2^A increased residence time, S156^ECL2^A and Q157^ECL2^A decreased the binding affinity, T256^6.58^A increased binding affinity and decreased residence time, and Y271^7.36^A prevented binding [134].

Other studies explored the dissociation process with a mechanistic perspective. Some GaMD simulations of NECA bound to the A_1_AR orthosteric site showed ligand dissociation in the absence of a PAM bound to ECL2, as anticipated in the section on allosteric modulation [117]. The dissociation process involved a pathway through ECL2 and ECL3.

In TAMD simulations accelerating the center of mass of ZMA out of A_2A_AR, contacts between the ligand and 16 residues in the upper receptor region were observed, especially in ECL2 and tip of helices TM2, TM6, and TM7 [134]. Twelve of those residues were mutated into Ala and, among them, E169^ECL2^A and Y271^7.36^A disrupted ligand binding, while two residue clusters affected the dissociation kinetics, with E169^ECL2^A, T256^6.58^A, and H264^ECL3^A increasing the dissociation rate, and I66^2.63^A, S67^2.64^A, and L267^7.32^A lowering it. These two clusters are topologically different; thus, a speculative interpretation of the binding process as multistep was provided. The first triad interacted with ZMA together with a structural water molecule in the initial system (like in the crystallographic structure with PDB code 4EIY [18]), and in the MD simulation the dissociation was preceded by the rupture of the interaction between E169^ECL2^ and H264^ECL3^ and by the loss of the triad-ligand interaction. An alternative binding intermediate during dissociation involved the ligand interaction with the second cluster, in a fashion that is similar to a different A_2A_AR-ZMA crystallographic structure, in which the E169^ECL2^-H264^ECL3^ bridge is broken (PDB code: 3PWH [15]).

The disruption of the salt bridge between E169^ECL2^ and H264^ECL3^ was also observed during the unbinding process of ZMA and LUF5452 from A_2A_AR through parallel tempering metadynamics simulations and, similarly, a bias had to be added to break the interaction between E172^ECL2^ and K265^ECL3^ to simulate the compounds’ dissociation from A_1_AR [135].

The influence of the E169^ECL2^-H264^ECL3^ salt bridge on the dissociation rate was also studied in order to rationalize the different A_2A_AR residence time of ZMA and four related ligands, whose X-ray structures showed a stabilizing interaction with H264^ECL3^ for compounds with longer residence time [19]. Metadynamics simulations were used to estimate the energy that is required to break the E169^ECL2^-H264^ECL3^ ionic bond, which was higher in the case of compounds with longer residence time.

Recently, the SuMD algorithm has been reversed and slightly modified to simulate ligand dissociation from its target [56]. The dissociation events of ZMA, adenosine, and NECA from A_2A_AR and adenosine from A_1_AR were simulated.

### 3.6. Water Contribution in Ligand-Binding

Water molecules have an important contribution in SBDD, together with the role they can play in modulating protein structure and function. In terms of contribution to ligand binding, in general, the binding site desolvation is one of the principal components of binding free energy, since water molecules are often entropically unfavored (limited degrees of freedom). However, there may or may not be an enthalpy compensation stabilizing the water molecule in the protein pocket, according to the protein environment. Water molecules can be classified as “happy” and “unhappy”, or respectively “cold” and “hot”, according to their stability on the protein surface as compared to the bulk solvent. The displacement of “happy” water molecules by a ligand is unfavored, with these molecules playing a structural role and mediating protein-ligand interactions; conversely, the displacement of “unhappy” water molecules, generally residing in hydrophobic environments, is favored, with consequent implications in SBDD [136,137].

Several computational methods have been developed for characterizing hydration sites on the protein surface. Among them, a method, called WaterMap, calculates the entropic and enthalpic components of water molecules through a statistical thermodynamic analysis of water clusters (hydration sites) obtained from MD simulations [138]. This tool was applied to characterize the hydration sites within the A_2A_AR binding site and analyze the SAR of a series of triazolylpurines that could not be explained by ligand-protein interactions, steric or desolvation effects. Small aliphatic substituents at the purine C2 position decreased the compound potency by occupying a region predicted to host stable water molecules. Longer aliphatic substituents increased the potency, because they extended to a region containing unstable waters, with a consequent compensation and favorable predicted binding free energy. The computed binding energies associated with water displacement correlated well with the experimental data [139].

Moreover, the identification and characterization of hydration sites is not merely an indicator for replacement, but it can be exploited to study possible water-mediated ligand–protein interactions. In fact, the inclusion of some explicit water molecules identified though WaterMap in a virtual screening applied to A_2A_AR increased the enrichment of active compounds over decoys [140].

**Figure 4:**
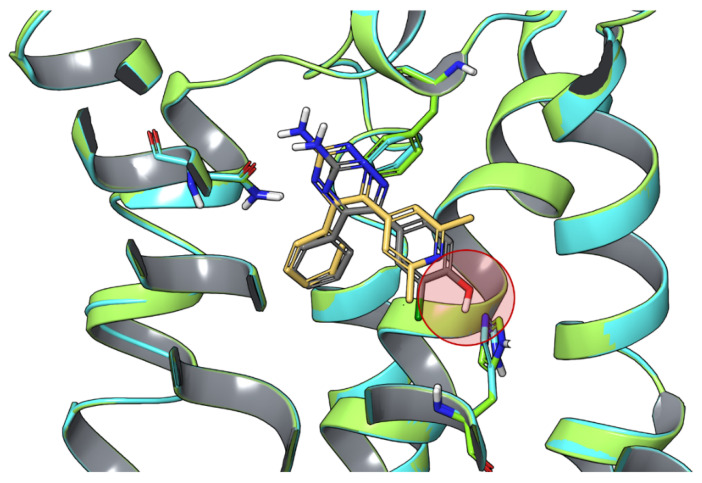
Superposition of the X-ray structures of A_2A_ AR bound to T4E (receptor in light green and ligand in gray; PDB ID: 3UZC [17]) and T4G (receptor in cyan and ligand in orange; PDB ID: 3UZA [17]). The area of “unhappy” water molecules favorably displaced by the hydroxyl moiety of T4E is highlighted by a red circle.

An analysis of the water hotspots in the binding site of the crystallographic compounds T4E and T4G, together with a series of 5,6-diaryl-1,2,4-triazines having different residence time, was performed with a pipeline, including WaterMap [17,141]. A low residence time characterized compounds with a benzene ring at position 6. In these cases, a hydration site with “unhappy” water molecules was observed between TM2, TM3, and TM7 and stabilized by H278^7.43^. The benzene ring at position 6 pointed toward the hotspot, and this could explain the low residence time of these molecules. Compounds with a *p*-hydroxy-benzene ring at position 6 had just one water molecule in the hotspot, but with no “unhappy” character and stabilized by an interaction with the hydroxy group, which could explain their increased residence time (Figure 4). The H-bond network was also stabilized by compounds with a pyridine at position 6, being characterized by an increased ligand residence time.

A similar rationalization was provided while analyzing the water network in the A_2A_AR with two other tools, employing the fluid dynamics properties of water (RMSF < 1.4Å during 200 ps) to identify hydration hot spots in the protein binding site and subsequently computing the water residence time (occupancy) in each hotspot. A first version projected the residence time in a two-dimensional (2D)-grid, called Water Fluid Dynamics (WFD) map [142]. The WFD of a ZMA-bound A_2A_AR identified high occupancy hotspots in proximity of Y9^1.35^, E13^1.39^, and H278^7.43^ mediating interactions with the ligand triazolotriazine core, and in proximity of N253^6.55^ and E169^ECL2^ mediating interactions with the compound exocyclic nitrogen, in good agreement with crystallographic water molecules. The same water network was found in presence of NECA and overlapped with the one obtained from a pseudo-apo simulation. A similar algorithm, AquaMMapS, was developed to make three-dimensional (3D) maps of hydration sites on the basis of the fluid dynamics properties of water [143]. Simulations and analysis of ZMA-bound and pseudo-apo trajectories gave results similar to those that were obtained by WFD. The binding pocket region at the ligand-TM1 interface was populated by stationary water molecules also during a SuMD trajectory of ZMA approaching to the receptor.

Another work introduced an alternative method to investigate water hot spots on protein surfaces from MD simulations [144]. In this protocol, most persistent waters on the protein surface are identified and then SMD is used to force hydration site desolvation, with the aim to identify regions that are easily depleted of water molecules, which are potentially the most advantageous to be filled by the ligand. This method was validated using A_2A_AR and the aforementioned triazolotriazine test set and showed good agreement with experimental data.

In a work cited in the previous paragraph, we have described the use of atomistic ensemble-based steered molecular dynamics to study the dissociation of 17 ligands from A_2A_AR [133]. In that study, a change in the water-ligand interaction energy (ΔE_LW_) between unbound and bound ligand states was computed during the simulations and correlated well with the compounds’ residence time, but not with their binding affinity. The rationale is that ligands with high ΔE_LW_ are badly hydrated in the bound state, so their hydrophilic interactions are buried, and this kind of interaction are known to increase the ligand residence time [145]. The derivatization of NECA with an arylalkyl extension at position C2 (CGS21680), and with an arylalkyl substituent at N^6^ in addition to an extended moiety at C2 (UK432,097) increased the residence time. Each of these substituents points toward the extracellular vestibule and they can prevent the entrance of water into the binding pocket.

A method combining SuMD, metadynamics, and suMetaD, enables the investigation of ligand binding and dissociation and retrieval of the transition state conformations of the two processes [146]. This algorithm was applied to A_2A_AR and six ligands, three xanthines (XAC, DPCPX, KW3902), and three triazolotriazines (ZMA, Z48, Z80), whose transition state thermodynamics appeared to be connected to the characteristics of water molecules solvating the binding pocket and the compounds. The transition state free energy was similar for all the compounds, but different enthalpic and entropic contributions were observed. In the case of binding, a transition state with ligand in the binding pocket was associated to a high enthalpic barrier (i.e., ZMA and Z80), due to the displacement of stable water molecules; while a transition state with ligand located in proximity of the vestibule was associated with a higher entropic barrier (i.e., XAC, KW3902, DPCPX, Z48), due to the water entrapped in the orthosteric site. Similarly, in the case of dissociation, transition states with high enthalpic barriers corresponded to a ligand in the orthosteric pocket (i.e., ZMA), while transition states with higher entropic barriers corresponded to all ligands in proximity to the vestibule.

## 4. Conclusions

The enhancement of computing power provided by new hardware and software technologies has been making MD simulations a helpful tool in molecular modeling and SBDD. The inclusion of protein flexibility and explicit solvent provides two advantages over molecular docking. Their inclusion enables the assessment of complex stability over time and can identify alternative binding mode(s) through induced fit phenomena, together with the interaction with structurally relevant water molecules. Moreover, the analysis of water molecules in explicit-solvent MD simulations might suggest chemical modification of ligands maximizing the replacement of thermodynamically unfavored water molecules and minimizing the replacement of favored waters.

New algorithms enhancing the classical MD simulations have enabled the exploration of long-timescale phenomena, like receptor activation, recognition, and dissociation pathways, filling the gap in experimental structural information necessary for investigating these processes at an atomistic level. This has implications in the drug discovery field, offering, for example, the possibility to rationally develop allosteric modulators or ligands focusing on optimized kinetics together with thermodynamic parameters.

## Figures and Tables

**Table 1:** Subtypes of adenosine receptors (ARs) and P2YRs and their characteristics.

Receptor Class	Receptor Subtype		G Protein Types	Endogenous Agonists
Adenosine Receptors (P1 Receptors)	A_1_		G_i_, G_o_	Adenosine
	A_2A_		G_s_, G_olf_	Adenosine
	A_2B_		G_s_, G_q_	Adenosine
	A_3_		G_i_	Adenosine, Inosine
P2Y Receptors	P2Y_1_-like	P2Y_1_	G_q_	ADP
		P2Y_2_	G_q_, G_i_	UTP, ATP
		P2Y_4_	G_q_, G_i_	UTP, ATP, GTP
		P2Y_6_	G_q_	UDP
		P2Y_11_	G_q_, G_s_	ATP
	P2Y_12_-like	P2Y_12_	G_i_	ADP
		P2Y_13_	G_i_	ADP
		P2Y_14_	G_i_	UDP-glucose, UDP, other UDP-sugars

**Table 2:** Reported AR and P2YR experimental structures (in the Protein Data Bank (PDB)) and their characteristics.

Receptor	Activation State	Ligand	Technique	PDB ID [Reference]
Adenosine Receptors (ARs)				
A_1_AR	Inactive	PSB36	X-ray	5N2S [24]
		DU172 (covalent)	X-ray	5UEN [38]
	Active (G_i_)	Adenosine	Cryo-EM	6D9H [39]
A_2A_AR	Inactive	ZM241384	X-ray	3EML [14], 3PWH [15], 3VG9 [16], 3VGA [16], 5UVI [22], 5JTB [23], 6AQF [27], 6MH8 [29]
		ZM241384, Na^+^ allosteric mod.	X-ray	4EIY [18], 5IU4 [19], 5K2A [20], 5K2B [20], 5K2C [20], 5K2D [20], 5NLX [25], 5NM2 [25], 5NM4 [25], 5VRA [26], 5OLG [28], 6JZH [30], 6PS7 [31]
		12b, Na^+^ allosteric mod.	X-ray	5IUA [19]
		12x, Na^+^ allosteric mod.	X-ray	5IUB [19]
		12c, Na^+^ allosteric mod.	X-ray	5IU7 [19]
		12f, Na^+^ allosteric mod.	X-ray	5IU8 [19]
		XAC	X-ray	3REY [15]
		Caffeine	X-ray	3RFM [15]
		Caffeine, Na^+^ allosteric mod.	X-ray	5MZP [24]
		Teophylline, Na^+^ allosteric mod.	X-ray	5MZJ [24]
		PSB36, Na^+^ allosteric mod.	X-ray	5N2R [24]
		T4G	X-ray	3UZA [17]
		T4E	X-ray	3UZC [17]
		T4E, Na^+^ allosteric mod.	X-ray	5OLZ [28], 5OM1 [28], 5OM4 [28]
		Tozadenant, Na^+^ allosteric mod.	X-ray	5OLO [28]
		Cmpd-1	X-ray	5UIG [21]
		LUAA47070, Na^+^ allosteric mod.	X-ray	5OLV [28]
		Vipadenant, Na^+^ allosteric mod.	X-ray	5OLH [28]
		AZD4635, Na^+^ allosteric mod.	X-ray	6GT3
	Intermediate	UK-432097	X-ray	3QAK [32], 5WF5 [35], 5WF6 [35]
		Adenosine	X-ray	2YDO [33]
		NECA	X-ray	2YDV [33]
		CGS21680	X-ray	4UG2 [34], 4UHR [34]
	Active (bound to mini-G_s_)	NECA	X-ray	5G53 [36]
		NECA	Cryo-EM	6GDG [37]
P2Y Receptors (P2YRs)				
P2Y_1_R	Inactive	MRS2500	X-ray	4XNV [40]
		BPTU	X-ray	4XNW [40]
P2Y_12_R	Inactive	AZD1283	X-ray	4NTJ [42]
	Intermediate	2MeSADP	X-ray	4PXZ [41]
		2MeSATP	X-ray	4PY0 [41]
